# Seeing through Events: Real-Time Moving Object Sonification for Visually Impaired People Using Event-Based Camera

**DOI:** 10.3390/s21103558

**Published:** 2021-05-20

**Authors:** Zihao Ji, Weijian Hu, Ze Wang, Kailun Yang, Kaiwei Wang

**Affiliations:** 1 National Engineering Research Center of Optical Instrumentation, Zhejiang University, Hangzhou 310058, China; jizihao@zju.edu.cn (Z.J.); huweijian@zju.edu.cn (W.H.); wangze0527@zju.edu.cn (Z.W.); 2 Institute for Anthropomatics and Robotics, Karlsruhe Institute of Technology, 76131 Karlsruhe, Germany; kailun.yang@kit.edu

**Keywords:** event-based camera, computer vision for visually impaired people, sonification, unsupervised object tracking

## Abstract

Scene sonification is a powerful technique to help Visually Impaired People (VIP) understand their surroundings. Existing methods usually perform sonification on the entire images of the surrounding scene acquired by a standard camera or on the priori static obstacles acquired by image processing algorithms on the RGB image of the surrounding scene. However, if all the information in the scene are delivered to VIP simultaneously, it will cause information redundancy. In fact, biological vision is more sensitive to moving objects in the scene than static objects, which is also the original intention of the event-based camera. In this paper, we propose a real-time sonification framework to help VIP understand the moving objects in the scene. First, we capture the events in the scene using an event-based camera and cluster them into multiple moving objects without relying on any prior knowledge. Then, sonification based on MIDI is enabled on these objects synchronously. Finally, we conduct comprehensive experiments on the scene video with sonification audio attended by 20 VIP and 20 Sighted People (SP). The results show that our method allows both participants to clearly distinguish the number, size, motion speed, and motion trajectories of multiple objects. The results show that our method is more comfortable to hear than existing methods in terms of aesthetics.

## 1. Introduction

According to the World Health Organization, there are currently about 253 million people worldwide with vision impairments [1]. It is difficult for Visually Impaired People (VIP) to visually feel the changes and appreciate the beauty of their surrounding environment. In order to help VIP see the surrounding environment, since the late 20th century, a variety of Electronic Travel Aids (ETAs) have been designed to convert information that are usually captured visually in the environment into information which can be received by other senses [2,3]. According to the National Research Council [4], the primary purpose of ETAs is to help VIP walk independently on the road. Thus, functions of ETAs include detecting objects in the environment that hinder VIP from moving forward, detecting pavement material, and so on. ETAs essentially help VIP recognize all kinds of stationary things around them. However, when Sighted People (SP) observe the world, eyes will be attracted first by moving objects [5]. Tracking and perceiving multiple moving objects is a function that most ETAs based on traditional image sensors cannot achieve.

Event-based cameras are exactly bio-inspired cameras mimicking the neural architecture of the eyes [6]. Instead of capturing fixed frames, they asynchronously compare the per-pixel brightness differences and output the corresponding event streams. Event-based cameras have many advantages: high dynamic range, low latency, and low power consumption [7], which are difficult problems in traditional visual tasks [8]. Based on these excellent characteristics, event-based cameras are widely applicated in object tracking and clustering. In this paper, we study in a setting where VIP are sitting or standing in a certain environment, so the camera remains still, and it allows us to investigate event-based moving object sonification design. Common algorithms used for event clustering of stationary cameras include Iterative Closest Point (ICP) algorithm [9], gradient descent [10], Mean-shift [11], Monte-Carlo methods [12], Gaussian Mixture Model [13], and particle filtering [14]. These algorithms all highlight the advantages of event cameras compared to traditional cameras. However, for assisting the perception in dynamic real-world scenarios, they still have certain problems, such as the number of clusters cannot be adaptive, and some algorithms require prior information about the perceived objects. With the in-depth research on event cameras, many works in recent years have focused on the event clustering method when the camera has ego-motion. In [15], the authors used the EM-like algorithm to jointly optimize the two parameters Motion Estimation θ and Event-cluster Likelihood Assignment P in the event 3d point cloud and generate the corresponding clustered motion-compensation event image. In [16], the authors obtained the completed clustering motion compensation map by minimizing the error function on time-image and maximizing event density on the event-count images. In [17], the authors incorporated the depth information of the scene compared to [15,16]. They used three encoder-decoder architectures in the neural network to extract the depth, pose, and motion information in the image. The most similar to our method is [13]. The difference between our method and the method in [13] is that when the number of clusters in the first frame is given, our method can adapt to the number of clusters in each video frame without manually specifying it. Thus, when an object enters, it is marked as a new cluster, and when an object exits, its corresponding cluster is deleted. The remaining clusters maintain the continuity between frames.

Sonification is a crucial method to convert sensing results into auditory data available to VIP. Early research sonified a picture by scanning the picture from left to right while binding elevation with pitch and brightness with loudness to represent its information [18]. However, the main disadvantage of this method is the redundancy of audio information, which makes VIP unable to easily distinguish the content of the scene while bearing a certain amount of auditory pressure. In order to solve above problems, in [19], the authors proposed to suppress the background to improve the sonification of objects of interest so that the sound information about the object is more concentrated and apparent, more accessible to be distinguished by VIP, and the overall auditory pleasure is improved. Currently, a bunch of research works are becoming increasingly more sophisticated, involving sonification methods of various elements of the picture, such as color [20], edge [21], texture [22], et al. [23]. In [24], the authors reviewed the existing sonification mapping strategies of physical characteristics. At the same time, the work of sonification system design based on the assistance of VIP has been increasing in recent years. The common point of these works is that they all include a sensor that captures the surrounding scene, an image processing method, a sonification method, and a sound output device [25,26,27,28,29,30]. VIP assists-oriented sonification methods for different subdivision tasks, like path sonification [31] and depth image sonification [32]. The most similar sonification method to this paper is the “Aquarium Series” [33,34,35]. They treated the fish in the aquarium scene as separate individuals and use multiple musical features to map the attributes of the fish. The MIDI (short for Musical Instrument Digital Interface) they used is either precomposed or randomly played major triads [34]. In our method, we use MIDI notes to represent the trajectory of the object’s movement and use the sonification method that is more easily perceivable by people. Even if multiple objects are sonified at the same time, people will feel comfortable in the auditory sense.

In this research, we propose a real-time sonification framework based on the event-based camera to help VIP understand the moving objects in the scene. To the best of our knowledge, we are the first to use the event-based camera as a sensor to obtain moving object data and use it for sonification to assist VIP in perceiving the surrounding environment. Compared to traditional image cameras, the event-based camera can significantly reduce the redundant information of the image data, reduce the complexity, and improve the speed and accuracy of subsequent image processing- and sonification algorithms. At the same time, VIP can pay more attention to changes in the surrounding environment, just like SP, instead of sticking to static objects without any changes.

**Figure 1:**
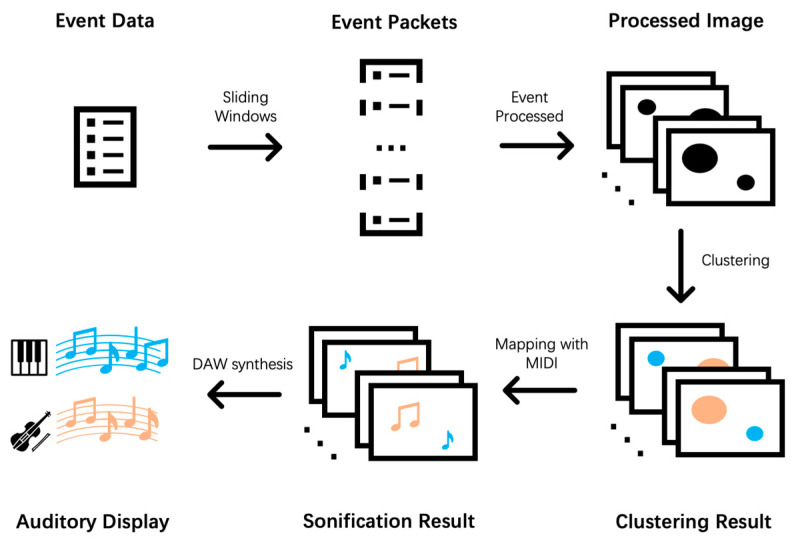
Flow chart of proposed sonification framework with the event-based camera.

We present a framework for VIP to perceive moving objects in the surrounding environment, as shown in Figure 1. We first use an event-based camera as the imaging sensor to collect event stream information of moving objects in the detected surrounding environment (assuming that the ambient light is constant). Then, we leverage an improved unsupervised learning algorithm based on the Gaussian Mixture Model (GMM) [36] to cluster the event stream. Finally, a sonification algorithm based on music theory [37] is employed to generate audio feedback of the clustered event stream. The output audio is then played to VIP through bone conduction headphones.

The main contributions of this paper are: Firstly, this work is the first to propose the issue that helps VIP understand multiple moving objects in the surrounding environment. Secondly, this paper proposes an event camera-based sonification framework to help VIP understand moving objects in the surrounding environment. The proposed framework is helpful for VIP to perceive moving objects in the surrounding environment easily and significantly enhance the acoustic comfort of VIP, which adds a whole new dimension to the VIP’s perception of the world. Thirdly, we conducted a user study to evaluate the quality of the sonification method from multiple dimensions for both SP and VIP and finally yielded a sonification method that is both artistic and practical. Moreover, we enrich environmental perception functions to the existing ETAs.

The rest of this paper is organized as follows. Section 2. describes our clustering and sonification methods in detail. Section 3. introduces the comprehensive experiments to test the scientificity and artistry of our sonification method. Section 4. presents the results and discussion of the experiments. Section 5. draws the conclusions.

## 2. Framework

In this section, we mainly introduce the entire framework from capturing moving objects with event-based cameras to mapping objects into sounds. In Section 2.1, we introduce how to track the moving objects represented by the event; Section 2.2 details the sonification method for each cluster; and in Section 2.3, we make a summary, presenting a full tracking and sonification framework.

### 2.1. Tracking Method

#### 2.1.1. Principle of Event-Based Camera

An Event-based camera is a pixel-level bionic camera that asynchronously responds to changes in light intensity within the field of view. Its output is called “event”, usually a four-dimensional array, including timestamp (one-dimensional), coordinates (two-dimensional), as well as polarity (one-dimensional). Specifically, we denote the logarithm intensity of the coordinate x=(x,y) in the field of view as L(x,t)=logI(x,t). When the change in intensity of a pixel $x_{k}$ reaches a threshold C (e.g., 10–15% relative change), the event camera will output an event in response to the change at that pixel, denoted as $e_{k}=\left(t_{k},x_{k},s_{k}\right)$. The following equation can express this intensity change:

(1) $$\begin{array}{c} \;\Delta L\left(x_{k},t_{k}\right)=L\left(x_{k},t_{k}\right)-L\left(x_{k},t_{k}-\Delta t_{k}\right)=s_{k}C \end{array}$$

where $t_{k}$ is the timestamp of the current event, $\Delta t_{k}$ refers to the time difference between the last event and the current event at the pixel $x_{k}$, and $s_{k}\in \left\{+1,-1\right\}$ represents the polarity of the current event.

#### 2.1.2. Problem Statement

According to the different application tasks, there are different representation methods to obtain meaningful interpretations [7]. This paper uses the “Events Packet” to process events. After processing, each packet can represent a traditional image frame. Specifically, given a space–time volume  V=Ω×T, Ω represents a two-dimensional image plane, and T represents time. Each volume contains one event packet $ℰ={\left\{e_{k}\right\}}_{k=1}^{N_{e}}$. Our task is to cluster the events in each event packet, which means that the events of the same object are divided into one cluster. At the same time, the number of clusters in each packet, that is, the number of objects is adaptive. The conditions in this task are: the ambient light intensity remains unchanged, and the event camera has no self-motion. This is an unsupervised task, which means there is no prior information about the objects being clustered.

#### 2.1.3. Adaptive Gaussian Mixture Model

In this paper, we use Gaussian Mixture Model (GMM) as our base model to cluster each event packet. The reasons for using GMM are listed as follows: Firstly, compared with other standard clustering algorithms (https://scikit-learn.org/stable/modules/clustering.html, accessed on 16 May 2020) (e.g., Affinity Propagation, MeanShift, Spectral Clustering, Ward, Agglomerative Clustering, DBSCAN, OPTICS, and BIRCH), under the same conditions, GMM with MiniBatch Kmeans has higher computational efficiency, which can meet the real-time requirement for assisting VIP; Secondly, the data distribution of event cameras is more suitable for GMM. GMM is a distance-based clustering method, which has an advantage in using distance (such as Euclidean distance) to distinguish different clusters. The event-based camera generates events asynchronously according to changes in light, among which events generated by the same object have a relatively close Euclidean distance. That is in line with the clustering principle of GMM. In addition, the use of GMM can also help roughly estimate the circularity of a cluster’s shape. Although it cannot represent the exact shape of most objects in the real world, the relative length and width of the object can be obtained, which we use as an object appearance information for sonification.

The GMM is a probabilistic model, which is a mixture model composed of multiple (multivariate) Gaussian distributions. Specifically, define x as a random vector in the n-dimensional space X, and x obeys Gaussian distribution, and its probability density function is:

(2) $$\begin{array}{c} \;p\left(x\right)=\frac{1}{{\left(2\pi \right)}^{\frac{n}{2}}{\left|\Sigma \right|}^{\frac{1}{2}}}e^{-\frac{1}{2}{\left(x-\mu \right)}^{Τ}\Sigma^{-1}\left(x-\mu \right)} \end{array}$$

where μ is an n-dimensional mean vector, and Σ is an n×n covariance matrix. Since the Gaussian distribution is completely determined by μ and Σ, we denote its probability density function p(x) as p(x|μ, Σ).

The mixed model is the basic model of GMM, which is a linear combination of multiple weighted probability density functions. Therefore, the probability density function of the Gaussian mixture model is shown in the following equation:

(3) $$\begin{array}{c} \;p_{ℳ}\left(x\right)=\overset{k}{\underset{i=1}{\sum }}\alpha_{i}\cdot p\left(x\;|\;\mu_{i}\;,\;\Sigma_{i}\right) \end{array}$$

where $\alpha_{i}$ is the mixture coefficient of the i-*th* Gaussian mixture component, $\overset{k}{\underset{i=1}{\sum }}\alpha_{i}=1$. Moreover, $\mu_{i},{\;\Sigma }_{i}$ are the mean vector and covariance matrix of the i-*th* Gaussian mixture component, respectively.

Now suppose that we have a set of unlabeled data $D=\left\{x_{1},x_{2},\ldots ,x_{m}\right\}$, and the goal is to find a GMM that best matches the dataset. In order to calculate all the parameters of this GMM, we use the Expectation-Maximization (EM) algorithm. The first step of the algorithm is Expectation, which uses the existing estimated value of the hidden variables to calculate the maximum likelihood (E step); The second step is Maximization, which maximizes the maximum likelihood estimation obtained at step E to calculate the value of the parameter (M step). The parameter estimation values calculated in step M are used in the next step E calculation, and this process is continuously alternated. Specifically, the application of the EM algorithm to calculate the parameters of the GMM includes the following two steps:

(1) E step. Let $z_{j}\in \left\{1,\;2,\;\ldots ,k\right\}$ denote the Gaussian mixture components of unlabeled data sample $x_{j}$, which is an unknown value. According to Bayes’ theorem, the posterior probability of $z_{j}$ is:
  (4) $$\begin{array}{c} \;\;p_{ℳ}\left(z_{j}=i|x_{j}\right)=\frac{\alpha_{i}\cdot p\left(x_{j}\;|\;\mu_{i}\;,\;\Sigma_{i}\right)}{\sum_{l=1}^{k}\alpha_{l}\cdot p\left(x_{j}\;|\;\mu_{l}\;,\;\Sigma_{l}\right)} \end{array}$$
(2) M step. According to the posterior probability calculated in the previous step, we use maximum likelihood estimation to obtain the new GMM parameters. The maximum (logarithmic) likelihood of GMM is shown in the following equation:
  (5) $$\begin{array}{c} \;LL=\overset{m}{\underset{j=1}{\sum }}\ln\left(\overset{k}{\underset{i=1}{\sum }}\alpha_{i}\cdot p\left(x_{j}\;|\;\mu_{i}\;,\;\Sigma_{i}\right)\right) \end{array}$$

We use the above equation to calculate the partial derivative of each parameter $\alpha_{i},\;{\;\mu }_{i},{\;\Sigma }_{i}\;$ and make them equal to 0, respectively. The new parameters can be obtained by the following three equations:

(6) $$\begin{array}{c} \;\alpha_{i}=\frac{1}{m}\overset{m}{\underset{j=1}{\sum }}p_{ℳ}\left(z_{j}=i|x_{j}\right) \end{array}$$

(7) $$\begin{array}{c} {\;\mu }_{i}=\frac{\sum_{j=1}^{m}p_{ℳ}\left(z_{j}=i|x_{j}\right)\cdot x_{j}}{\sum_{j=1}^{m}p_{ℳ}\left(z_{j}=i|x_{j}\right)} \end{array}$$

(8) $$\begin{array}{c} {\;\Sigma }_{i}=\frac{\sum_{j=1}^{m}p_{ℳ}\left(z_{j}=i|x_{j}\right)\cdot \left(x_{j}-\mu_{i}\right)\cdot {\left(x_{j}-\mu_{i}\right)}^{Τ}}{\sum_{j=1}^{m}p_{ℳ}\left(z_{j}=i|x_{j}\right)} \end{array}$$

The above two steps keep looping until the termination condition is finally reached, including the maximum number of cycles or reaching the likelihood improvement threshold.

The algorithm designed in this paper is used for clustering of event streams of any length of time. Therefore, the following problems still need to be overcome when using the GMM model. First, the problem of continuity between the upper and lower frames. That is, if the object in the previous frame appears in the next frame, the GMM model needs to mark the events representing the object as the same cluster both in the upper and lower frames instead of just considering the relationship between events in a single frame. Second, the GMM model is an algorithm that requires a priori number of clusters. However, this is not suitable in perceiving multiple moving objects in the scene, and the algorithm needs to calculate the number of clusters (that is, the number of objects) of the current scene in real-time. Based on the above two issues, we propose the Adaptive Gaussian Mixture Model for the VIP-assisted sonification method based on event data.

Firstly, we use the sliding window method to split an entire event stream into several packets. The time interval of each event packet is the same. That is, the window size is constant. The step size equals the window size, which means no overlap of events between two adjacent event packets. We divide the relationship of two adjacent event packets into three types. Firstly, these two packets contain the same object (object constant). Secondly, a new object enters the latter packet compared to the previous one so that events are representing a new object in the latter packet (object entered). The third is that objects in the latter packet exit or stop compared to the previous packet which means that the events of these objects have disappeared (object exited).

For object constant, we save the weights, means, and covariance of the GMM model used by the previous event packet and use them to initialize the GMM model used by the current event packet.

For object entered and object exited, we first use per-sample average log-likelihood (score (https://scikit-learn.org/stable/modules/generated/sklearn.mixture.GaussianMixture.html, accessed on 16 May 2020)) to determine whether the number of clusters is changed. When the score of the current packet is higher than the score of the previous packet by a percentage threshold, we consider the number of clusters to change. This percentage threshold is a hyperparameter, which can vary according to different datasets. In this paper, we set the percentage threshold to 20%, obtaining the best accuracy in this scenario. Secondly, if the weight of one or several Gaussian mixture components in the current GMM is 0, it means that objects are exiting. Correspondingly, we will re-initialize a GMM with random values and set the number of clusters to that used in the previous packet minus the number of exit objects. Otherwise, we consider that an object has entered. To ensure the continuity between the frame detected by the initialized GMM model and the previous frame, we use the Euclidean distance of the cluster center point and the number of events in the cluster to judge the consistency of the clustering of the upper and lower frames. Note that the case of multiple objects entering is beyond the scope of this paper due to the need for additional detection algorithms. Similarly, we re-initialize a GMM with random values and set the number of clusters used in the previous packet plus one. We assume that the number of clusters of the first packet in the entire event stream is known.

#### 2.1.4. Tracking Pipeline

Now, we introduce the proposed entire object tracking pipeline. In the first step, we split the entire event stream into several event packets through the sliding window method. In the second step, for each event packet, we convert it into a binary image frame. The height and width of the image frame are respectively the height and width of the imaging field of view of the event camera. For each coordinate in the image frame, when there are one or more events existing, we mark the coordinate as white, otherwise black. In the third step, we use the median filter algorithm with kernel size = 5 to eliminate the salt-and-pepper noise introduced by the event camera and re-save the resulting image using the event packet format. Since the task in this paper is only to track the object, it is not necessary to use the polarity attribute of each event. In the fourth step, we cluster each event packet using the adaptive GMM to obtain the category label of each event and output the labeled video.

### 2.2. Sonification Method

The sonification technique used in this paper is called “parameter mapping sonification” [38]. We aim to design a set of sonification methods for the continuous movement of objects, expressing as much information as possible while making the auditory display sound more comfortable and pleasant. According to [39], applying music theory knowledge and performance skills in music makes the auditory display more enjoyable and allows the audience to accept it to the greatest extent. In this work, we use Western music theory for sonification, which uses discrete sounds to represent continuous data. This way of alternatively using sounds and intervening silence to represent data will make the overall auditory sense neither too obtrusive nor too habituating, which is a design method that combines art and science [38].

#### 2.2.1. Musical Instrument Digital Interface (MIDI)

In order to manipulate the most comprehensive music parameters, we use MIDI to record music. In this paper, we use parts of the MIDI components, which consists of instrumental tracks containing a series of notes and control change messages.

MIDI notes contain four attributes: start, end, pitch, and velocity. The first two attributes, start and end, indicate the time when a note appears and disappears. By controlling the end and start of the same note, we can not only define the length of the note but also set up the density of a bunch of notes so that people can get rush or soothing music pieces while keeping the MIDI tempo attribute unchanged. The third attribute pitch is the discrete frequency. The larger the pitch, the higher the frequency corresponding to the note, and vice versa. The frequency of each pitch follows Western music theory. This work uses the default twelve equal temperaments. The fourth attribute velocity is discrete loudness—the greater the velocity, the louder the note. The value of velocity is an integer from 0 to 127.

MIDI control change messages convey positional information to control various functions in the synthesizer [40]. There are currently 120 controller numbers, and only two of them are used in this work: No. 10 Pan and No. 64 Pedal. The Pan is used to conveying stereo audio with an integer ranging from 0 to 127. The smaller the value, the sound component in the left channel is louder, and vice versa. The Pedal is the sustain pedal in the piano, which can increase the sustain of the corresponding note.

MIDI instrumental tracks are the container of the MIDI notes and control change messages. Both of them can be saved in instrumental tracks. The primary function of instrumental tracks is to specify which instrument to be used in the track. Typically, only 128 instruments defined by the General MIDI Specification can be specified. However, this work uses Digital Audio Workstations (DAW) to mount sub-track timbre for more musical auditory display.

#### 2.2.2. Mapping Method

The method of using MIDI to represent moving objects is introduced in detail as follows.

First, we map each object that appears in the scene into different MIDI instrument tracks. This is “one-to-one mapping”, so there is no one instrument track representing two objects, while there is no one object represented by two instrument tracks.

Second, for each moving object, we use a series of notes, control change messages, and instruments to represent the appearance and motion attributes. In this work, the appearance attributes specifically refer to the size of the objects, and the motion attributes specifically refer to the trajectory and speed of the objects. Table 1 lists the mapping relationship between object attributes and MIDI attributes. Note that there is an extra new appearance attribute that will be introduced in subSection 2.2.3.

(1) Size. We mainly distinguish the relative size of objects based on the timbre. Larger objects use the pad timbre, which usually has a longer attack time and a longer release time [41]. The overall auditory sense is continuous and deep. Smaller objects use the lead timbre, which usually has a shorter attack time and a longer sustain time. Lead always makes auditory sense clear, sharp, and feels a strong sense of crispness.
(2) Speed. The speed of an object is a relative concept. In different scenarios and applications, the definition of fast (slow) speed is different. This work focus on using the sonification method to distinguish between speed types, so the specific value of speed is not the focus of the study. We simply set a speed threshold $v_{s}$. When the object’s speed is greater than $v_{s}$, the corresponding note of the object appears every 75 ms; that is, the notes are denser. When the object’s speed is less than $v_{s}$, a note appears every 150 ms; that is, the notes are sparser.
(3) Abscissa. We linearly map the abscissa of the image plane to the pan value from 0 to 127, as shown in the following equation:
  (9) $$\begin{array}{c} \;p\left(w\right)=\lfloor \frac{127}{w_{c}}w\rfloor \end{array}$$
  where $w_{c}$ represents the width of the image plane, p(w) represents the pan value, and w represents the abscissa of the object. The abscissa of the object is the average of all events that make up the object. The pan value calculated by the Equation (10) is the MIDI note pan value generated from the object.
(4) Ordinate. We linearly map the ordinate of the image plane to two octaves from C4 (pitch = 60) to B5 (pitch = 83), as shown in the following equation:
  (10) $$\begin{array}{c} \;p\left(h\right)=\lfloor \frac{23}{h_{c}}h+60\rfloor \end{array}$$
  where $h_{c}$ represents the height of the image plane, p(h) represents the pitch, and h represents the ordinate of the event. The ordinate of the object is the average of all events that make up the object.

#### 2.2.3. New Attributes

As known to all, instruments are a prerequisite for each track to be played. Therefore, when employing different instruments to represent different objects, we aim to use different timbre attributes to represent more appearance attributes of the object. For example, as mentioned above, we use different instruments to represent the size of objects, lead for big objects, and pad for small objects. In addition to size, we further consider superimposing new timbre attributes on existing instruments to represent new attributes of objects. This new attribute needs to make the audio change as large as possible compared to the original audio. Thus, we experiment as introduced in Section 3.3, and we reach the following conclusion: Different musical instruments have different timbre attributes that make the most changes in audio. For lead, we use polyphony and monophony to distinguish different object attributes. For pad, we use the pedal to distinguish different object attributes. However, when the above changes are added, the audio is more complicated, and the accuracy of understanding the true attributes of the object is reduced. The above findings will be explained in detail in Section 3 and Section 4. Thus, in the end, to ensure that people can well perceive every attribute, the sonification method of this work only contains the four attributes of the object: Size, Velocity, Abscissa, and Ordinate. Moreover, we can also distinguish different objects through types of instruments.

### 2.3. Whole Framework

The whole framework is shown in Figure 1. We first use the event camera to record moving objects in the scene for a certain period to obtain an event data file. Second, we use the sliding windows method to divide the event into event packets of the equal time intervals. These event packets are processed as described in Section 2.1.4, and the corresponding image frames are output. Each frame of image is clustered using the Adaptive GMM algorithm, and the category label of each event is obtained and displayed on the image frame in different colors. At this time, the video connected by each frame of the image can use the sonification method mentioned in Section 2.2 to generate the corresponding MIDI file. Finally, we import the MIDI file into DAW and output the waveform file in .wav format with the corresponding instrument of each track, which is the auditory display of the moving objects in the scene.

## 3. Experiment Settings

This paper uses multiple MIDI attributes to display multivariate data. In order to verify that the sonification method is both scientific and artistic, we performed comprehensive experiments with both SP and VIP. In addition to self-comparison, we also compared our method with existing sonification methods to show the superiority of our method in representing multi-object data. In this section, we introduce our experiment settings in detail, including the datasets, the selection of participants, and the design of questionnaires.

### 3.1. Dataset

Most open-source event-based vision datasets are designed for computer vision tasks, such as optical flow estimation, post estimation, simultaneous localization, etc. The characteristics of the datasets for the above tasks are as follows:

(1) Datasets contain scene events due to camera movement.
(2) The scene is relatively complex, such as the number of objects is large, the shape of objects is complex, and objects move in three-dimensional space.

**Figure 2:**
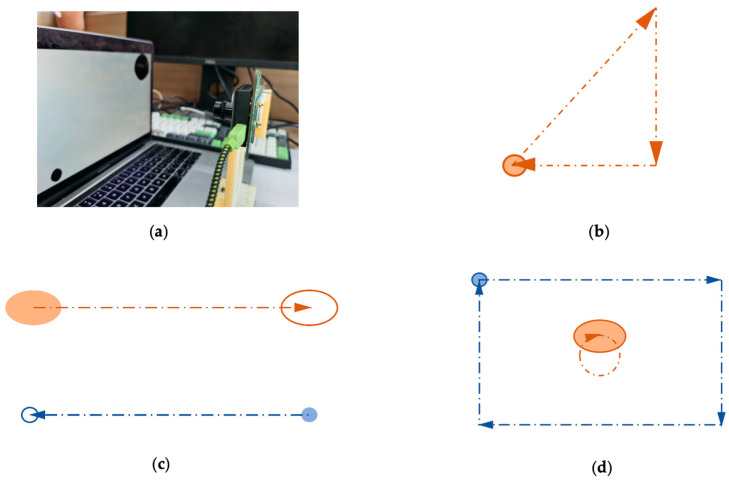
(**a**) Physical picture of using event camera to record the laptop screen; (**b**–**d**) are all animated slides of PowerPoint that make up the dataset. The solid circle represents the starting point of the object’s motion, the hollow circle represents the endpoint of the object’s movement, and the dotted line represents the trajectory of the object’s movement. Different colors represent different objects.

Moreover, there is no dataset specifically for sonification in the open-source event-based vision camera datasets. Therefore, in order to verify the effectiveness of our method, we build our dataset. We use PowerPoint’s animation function to simulate the two-dimensional translational movement of objects. More importantly, we can customize the appearance, motion properties, and number of objects, which is consistent with our original intention of the sonification design. We design a total of two sets of slides, one for training and the other for testing. The training set is used to inform the participants of the mapping relationship between the attributes of the objects and the sound, such as the size, quantity, movement speed, and movement trajectory. The testing set is used to test whether participants can understand the mapping relationship between audio and moving objects. We play two slides on the laptop and use an event camera to record the laptop screen. Our event camera SEEM1 comprises a Silicon Eye Event Sensor and a 9-DoF IMU. Our laptop is MacBook Pro (13-inch, 2017). The event camera is placed 30 cm in front of the laptop screen. Figure 2 shows the process of using an event camera to record the laptop screen with animation examples.

### 3.2. Participants

Twenty VIP volunteers from a blind school and 20 SP volunteers were invited to participate in the experiment. All participants have received complete primary education and can use computers skillfully. There are ten males and ten females in each category of participants. All the volunteers are in the 13–26 age range. It needs to be noted that we obtain the consent of the blind school to conduct the experiments.

### 3.3. Pre-Experiment

As mentioned in Section 2.2.3, we only perform sonification on the object size for object appearance attributes. However, we hope to ensure that the sonification method is both practical and artistic, which can represent more appearance information of multiple objects. Subsequently, we plan to add a new timbre attribute to represent a new appearance attribute of the object. We design an experiment to figure out what kind of timbre attribute makes the auditory sense significantly different. In this experiment, four commonly used MIDI Control Change (CC) messages are selected to modulate timbre. The experiment also uses polyphony instead of monophony as one of the timbre attributes. The specific alternative method is: for each note, generate a major triad with this note as the third note. The complete timbre attributes used in the experiment are listed as follows in Table 2.

We randomly select a video of double objects (one large and one small) from the dataset described in Section 3.1 and use the sonification method described in Section 2 to generate the corresponding MIDI. The MIDI contains two tracks, the big one with the lead instrument and the small one with pad instrument. Both tracks are monophonic. For each experimental group, we superimpose one of the attributes on one of the tracks and keep the other track unchanged. In the end, we obtain a total of ten experimental groups. The original MIDI, whose two tracks have no superimposed attributes, is regarded as the control group. The experimental group and the control group are shown in Table 3.

We ask all participants firstly to listen to these 11 MIDIs and then select one MIDI from experimental group 1 and the other MIDI from experimental group 2 that have the most significant difference in auditory sense from the control group. Thus, we obtain MIDI selection rates (=selected number/total number of people) of all experimental groups, as shown in Table 4.

From Table 4, we find that for different instruments, the timbre attributes that cause significant differences in an auditory sense are not the same. For the lead, more people think that polyphony has a significant difference, while for the pad, the pedal causes a more significant difference. Therefore, in the follow-up comprehensive comparison experiment, we further add an experimental group. The experimental group inherits all the features of the original control group and adds a new object feature using the pedal for large objects and polyphony for small objects. More details will be explained in Section 3.5.

### 3.4. Training

In order to make the participants fully understand our sonification method, we make an explanation video. In this video, we use several cases to illustrate our sonification method for moving objects. For each case, we first play the audio of sonification, then explain the above audio, and finally play the video with audio of the object’s movement. The first case explains the shape attributes of objects, using different timbres to represent different objects. The second case explains the density of the note, which is positively related to the speed of the object. The third case explains the direction of the object’s movement. The left and right channels indicate the horizontal movement of the object, and the pitch indicates the vertical movement of the object. The fourth case explains the movement of multiple objects, and it is combined with the previous cases to make multiple audios. Thereby, the subjects can better understand our sonification method and the mapping principles.

The whole video is about 5 min long. For SP, the video is played directly. For VIP, when it comes to watching the video, the experimenter helps the VIP understand the meaning of object motion in the video through touch.

### 3.5. Experimental Process

In this paper, we have designed a comprehensive experiment to prove the artistry and practicality of our sonification method. For evaluating the sonification method proposed in this paper, we have designed six dimensions, including object attributes (size, number, and new appearance attributes), motion attributes (trajectory and speed attributes), cocktail effect, perceived difficulty, audio comfort, audio, and motion adaptation. For these six dimensions, we have designed subjective and objective questions to increase the accuracy and rigor of the assessment. For any participant involved in the comprehensive experiment, they do not need to study the sonification method for a long time but only need about 5 min of video learning provided by the experimenter, as shown in Section 3.4. None of the participants in the experiment had a musical foundation, which ruled out the possible influence of musical prior knowledge on the experiment. We have carefully designed fourteen test videos, including eight single-object videos and six dual-object videos. Although all objects only contain translational motion, the combination of translational motion trajectory and speed is unique for each video. At the same time, to prove the advanced nature of our method, we also compare in with existing methods.

We set up a total of three groups for comparative experiments, as shown in Table 5.

The three groups include a control group and two experimental groups. The sonification method of the control group followed Section 2.2.2, including all motion attributes and one appearance attribute (size). The first experimental group has been added a new appearance attribute based on the control group. In the actual experiment, we set the new attribute to the width of the object. In other words, when the object is relatively wide, the large object will trigger pedal, and the small object will trigger polyphony, as mentioned in Section 2.2.3. The purpose of the first experimental group is to explore the multi-dimensional impact of adding a new object attribute on people’s access to information from the audio. At the same time, it allows us to explore the balance between the amount of information and the artistry of audio. The second experimental group is based on a previously published sonification method [3]. In order to reproduce the “Image Sonication” in their work, we convert the input gray video into depth video, which can be realized by setting the depth value D to 1 m. The three groups will share the following experimental settings.

We integrate all the experimental procedures into one video by following the training procedure mentioned in Section 3.4. The whole video contains 14 cases of object movement. At the same time, we prepare a questionnaire. For each case in the video, the participant needs to answer eight questions, including two objective questions and six subjective questions. The subjective questions are rated on 7-point Likert scales ranging from 1 (strongly disagree) to 7 (strongly agree). We list all the contents of the questions in the following.

Objective questions:

Q1: (Object Size) Multiple choice questions. There are three options in total: A. Objects are all large; B. Objects are all small; C. One large and one small. There are no more than two objects in all video cases. Therefore, when there is only one object, choose from option A and B; when there are two objects, choose from option A, B, and C.

Q2: (Object Number) Multiple choice questions. There are two options in total: A. One object; B. Two objects. Just choose the number of objects one have already heard.

Q3*: (New appearance attribute) Multiple choice questions. As mentioned above, we set the new attribute to the width of the object. Thus, the three options are: A. Objects are all wide; B. Objects are all narrow; C. One wide and one narrow. Attention, only the first experimental group has to answer this question, while the other two groups do not.

Subjective questions:

Q1: (Motion speed perception) I can well perceive the speed of all objects.

Q2: (Trajectory perception) I can well perceive the trajectory of all objects.

Q3: (Cocktail party effect) It is easier for me to recognize the attributes of one object than two (Only in the situation of multiple objects, the participant has to answer this question).

Q4: (Perception difficulty) I can easily know how objects move.

Q5: (Audio comfort) I think the audio is comfortable and will not feel irritable.

Q6: (Audio and motion adaptation) I think the audio is a good representation of the existing objects.

In each video case, participants need to complete the following in order. Firstly, listen to the audio corresponding to the video case. Secondly, answer the objective questions, and remember the speed and trajectory of the objects. Thirdly, watch the video case. Finally, answer the subjective questions. In particular, as VIP cannot see the movement of objects, we describe them in a tactile way. Moreover, we will help VIP record the answer to each question. Because it is difficult to measure the motion speed and trajectory of objects quantitatively, we regard the evaluation of movement speed and trajectory as a subjective test.

Because of the difference in the experimental population, the experiment is divided into two parts, SP attend online, and VIP attend offline in the School for the Blind. In this school, the experiment was carried out in the computer classroom. We explained the experimental process for the VIP. Firstly, we asked them to listen to the explanation video and help them fully understand it. Then we asked them to listen to the test video and cooperate with them to complete the test questionnaire.

## 4. Results and Discussion

In this section, we show the results of our experiments. Section 4.1 gives an analysis of the scores of each question in the control group. Section 4.2 and Section 4.3 give the comparative results of experimental group 1 and experimental group 2 with the control group respectively to analyze the advantages of different sonification methods.

### 4.1. Control Group Results

**Figure 3:**
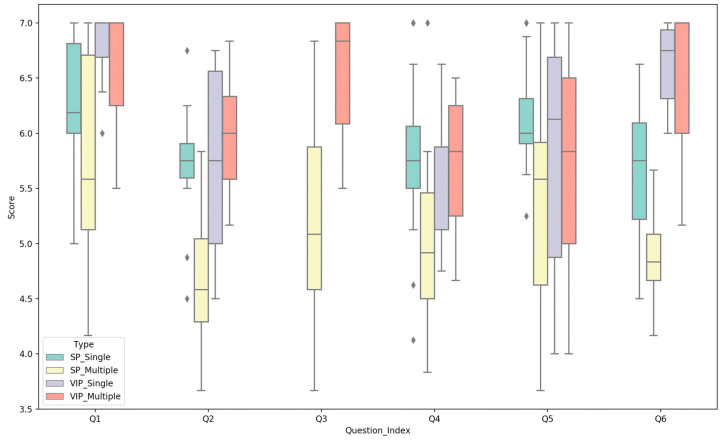
Box chart of subjective questions in different conditions in the control group. Q1 to Q6 indicate six subjective questions for evaluating motion speed perception, trajectory perception, cocktail party effect, perception difficulty, audio comfort, audio and motion adaptation. The score is based on the 7-point Likert scale, where 1 means strongly disagree and 7 means strongly agree.

The experimental results of the objective questions of the control group are shown in Table 6, and the subjective results are shown in Figure 3. From the overall results of the experiment, the score of VIP is generally higher than that of Sighted People (SP). According to the experimental results of objective questions and subjective questions Q1 and Q2, VIP have a significant advantage over SP for using audio to recognize the appearance and motion attributes of objects. In terms of the matching of audio and objects, VIP found the two better matches than SP did. This proves that our sonification method can accurately represent moving objects and is well perceived by people, whether VIP or SP.

We also notice that the correct rate of VIP for multi-object size attribute recognition is slightly lower than SP, but VIP’s cocktail effect score is much higher than SP. We can safely conclude that VIP can obtain the critical information more efficiently than SP when they hear multiple audio segments. That is, for a segment of audio that represents an object, this information will be more accurately and efficiently captured by VIP.

Regardless of the different participants, let us analyze the difference between single-object and multi-object scores. The scores of multiple objects are generally lower than that of a single object. This is because the amount of audio information is proportional to the number of objects, but the amount of information that people can perceive simultaneously is limited. However, in this experiment, we find that nearly all the indicators of multiple objects did not fall too much than those of a single object, and the average score of each subjective question is above four points, which proves that the results of each question are positive.

### 4.2. The Experimental Group 1 Results

The objective and subjective results of the experimental group 1 and 2 with the control group are shown in Table 7 and Table 8 respectively. In this subsection, we analyze the results of the experimental group 1.

In the objective questions, the correct rate of the control group in Multiple_Q1, Single_Q2, Multiple_Q2 are all higher than that of experimental group 1. As for Single_Q1, the results of the two groups are very close. The two experiments are based on the same sonification method, while the only difference is that experimental group 1 has been added an auditory dimension. Therefore, additional dimensions used to represent object attributes will significantly reduce people’s perception of other object attributes, except for the number of a single object. For Single_Q3 and Multiple_Q3, the correct rate of the two is also low, and Multiple_Q3 is only 5.6% higher than the correct rate of random selection (33.3%). This means that the amount of information that people can obtain from multiple complex attributes is limited. The sonification of the three object attributes exceeds the range of people’s ability to distinguish information.

In subjective questions, since Q3 is mainly used to study whether a sonification method has a cocktail effect rather than comparing two sonification methods, Q3 is canceled in the comparative experiment.

From Table 8, we can see that, except for Q5, the scores of the remaining questions of the control group are all higher than those of the experimental groups. Therefore, even though two groups are based on the same sonification method, adding an object attribute will still hurt the perception of audio. The difference between the scores of each question of the two groups is less than 0.5, which proves that adding an object appearance attribute has no significant impact on other dimensions such as object motion attributes, object perception difficulty, audio comfort, audio, and motion adaptation. At the same time, all the scores of the experimental group 1 are greater than four points, which together with the results of the control group proves the scientific and artistic nature of our proposed method.

### 4.3. The Experimental Group 2 Results

In this subsection, we analyze the results of experimental group 2.

In objective questions, the correct rate of experimental group 2 in each problem is not as good as that of the control group. Moreover, we also find that for Q1, the correct rate of a single object is lower than that of multiple objects. This is the only question in all groups where the correct rate of a single object is lower than that of multiple objects. The reason for the above two phenomena is that we use two completely different mapping principles. Hu et al.’s method [3] is based on a dense-pixel sonification method; that is, each non-black pixel in the video is mapped into a kind of sine wave, and all sine waves in each frame of the image are sounded at the same time. Although this method can represent all image pixel information, it is difficult for people to distinguish the appearance attributes such as the number and size of objects.

In subjective questions, we find that the scores of each question in experimental group 2 are lower than those in the control group. Especially in the case of multiple objects, the scores of the Q2, Q4, and Q6 questions do not exceed four points. This shows that when multiple objects are in motion, it is difficult for people to correctly perceive object’s trajectory through Hu et al.’s method, and they also think that the audio does not match the motion of the object. At the same time, because Hu et al.’s method only uses sine wave timbre and does not explicitly choose the instrument, listening to this audio for a long time will reduce people’s audio comfort. Our method considers the harmony between the timbres of different instruments, so when multiple objects appear, multiple instruments playing simultaneously will make people feel very comfortable in hearing sense.

### 4.4. Discussion

This subsection mainly discusses the scalability and robustness of our sonification method based on the above experimental results.

First, the algorithm used in this paper is based on the assumption that the camera is stationary. Nevertheless, works in [15,16,17] use different methods to recognize events generated by the camera’s ego-motion. The above algorithms are not used in this paper because the clustering algorithm that includes the ego-motion of the camera will consume more computing resources, and the sonification algorithm of each frame will not be able to achieve a real-time computing speed. In addition, the accuracy of this type of algorithm is not very high, which is a problem that has not been perfectly solved. However, the sonification method in this paper takes into account the camera moving. The change at this time is that the speed used for sonification mapping is the relative speed of the moving object and the camera, which is also in line with the SP’s usual perception of the world. Therefore, the sonification method designed in this paper can also be well extended to event processing algorithms that include the camera’s ego-motion.

Second, we did not use image data containing depth information. However, this has also been taken into account in our sonification method. In the design of the entire mapping method, we did not use the volume dimension. The volume dimension is usually mapped to depth in scene mapping [23]; that is, the distance between the object and the camera. When the object is closer to the camera, the volume is louder, and vice versa. Therefore, the sonification method in this work can also be appropriately extended to 3D scenes.

## 5. Conclusions and Future Work

In this paper, we have proposed a real-time sonification framework for multiple moving objects in the scene. We have verified in experiments with different types of people, including VIP and SP, that our method allows people to easily and accurately perceive the appearance and motion attributes of multiple objects, which is the scientific nature of our method. At the same time, our method is also artistic, as it not only makes people feel comfortable in the sense of hearing but also the audio can be well-matched with the various translational motions of the object. We also demonstrate through comprehensive experiments that the number of appearance attributes should be limited in a particular range. Although the use of timbre changes can map an infinite number of object appearance attributes in theory, the dimension of timbre that people can perceive is limited. At the same time, we also compare our method with state-of-the-art sonification methods. It demonstrates that our method is superior to the existing methods in terms of scientificity and artistry.

In the future, we aim to study the sonification method of moving objects when the camera contains self-motion. Meanwhile, we can add depth information to event data for sonification. We also have the intention to explore how to avoid the noise caused by a large number of musical instruments playing simultaneously when there are more objects in the scene.

## Figures and Tables

**Table 1:** The mapping relationship between object attributes and MIDI attributes.

		Note Pitch	Note Density	Pan	Pedal	Instrument	Poly-Phony
Appearance attributes	Size					√	
	New attribute				√		√
Motion attributes	Speed		√				
	Abscissa			√			
	Ordinate	√					

**Table 2:** The number and name of all Control Change (CC) messages used by pre-experiments.

Attributes No.	1 (CC #1)	2 (CC #64)	3 (CC #91)	4 (CC #93)	5
Attributes Name	Vibrato	Pedal	Reverb	Chorus	Polyphony

**Table 3:** The number and specific content of the control group and each experimental group.

Experimental Group 1 No.	Description: Lead with New Attributes	Experimental Group 2 No.	Description: Pad with New Attributes	Control Group
1-1	Lead with vibrato	2-1	Pad with vibrato	monophonic with no new attributes
1-2	Lead with pedal	2-2	Pad with pedal	
1-3	Lead with reverb	2-3	Pad with reverb	
1-4	Lead with chorus	2-4	Pad with chorus	
1-5	Lead with polyphony	2-5	Pad with polyphony	

**Table 4:** The selection rates of each experimental group.

	1	2	3	4	5
Experimental Group 1	30.0%	10.0%	0	0	60.0%
Experimental Group 2	22.5%	72.5%	0	0	5.0%

**Table 5:** The experimental settings of the control group and the experimental group included the sonification method and appearance attributes of each group.

	Sonification Method	Appearance Attributes Contained
Control Group	Ours	Object Size
Experimental Group 1	Ours	Object Size and Object Width
Experimental Group 2	Hu [3]	Object Size

**Table 6:** The correct rate of objective questions in different conditions of the control group. SP indicates Sighted People; VIP indicates Visually Impaired People. Single and Multiple indicate the number of objects. Q1 and Q2 indicate the index of questions for perceiving the number and the size of objects.

Objective Questions	Single_Q1	Multiple_Q1	Single_Q2	Multiple_Q2
SP	99.0%	98.6%	94.8%	80.6%
VIP	100.0%	100.0%	96.4%	78.6%

**Table 7:** The correct rate of objective questions in different conditions of all the groups. Q1 to Q3 indicate three objective questions for perceiving object size, number, and width, respectively.

Objective Questions	Single_Q1	Multiple_Q1	Single_Q2	Multiple_Q2	Single_Q3	Multiple_Q3
Control Group	99.5%	99.3%	95.6%	79.6%	/	/
Experimental Group 1	100.0%	72.2%	88.9%	55.6%	72.2%	38.9%
Experimental Group 2	66.7%	77.8%	61.1%	44.4%	/	/

**Table 8:** The 7-point Likert scales-based scores of subjective questions in different conditions of all the group.

Subjective Questions		Q1	Q2	Q4	Q5	Q6
Control Group	Single	6.509	5.72	5.684	5.938	6.142
	Multiple	6.175	5.314	5.371	5.54	5.671
Experimental Group 1	Single	6.111	5.611	5.556	6.278	5.722
	Multiple	5.667	4.944	5.056	6.111	5.333
Experimental Group 2	Single	5.667	4.556	4.833	5.833	5.111
	Multiple	4.833	3.611	3.722	5.111	3.833

## Data Availability

Not applicable.
